# Characterization of a Luminescence-Based Serum Bactericidal Activity Assay for Human Sera Against a Panel of *Salmonella* Strains

**DOI:** 10.3390/microorganisms13122757

**Published:** 2025-12-04

**Authors:** Maria Grazia Aruta, Luisa Massai, Daniele De Simone, Federica Boretto, Marta Benincasa, Miren Iturriza, Martina Carducci, Francesca Mancini, Rocio Canals, Simona Rondini, Omar Rossi

**Affiliations:** GSK Vaccines Institute for Global Health (GVGH), GSK Global Health Vaccines R&D, Via Fiorentina 1, 53100 Siena, Italy; maria-grazia.x.aruta@gsk.com (M.G.A.); luisa.x.massai@gsk.com (L.M.); daniele.x.desimone@gsk.com (D.D.S.); federica.x.boretto@gsk.com (F.B.); marta.x.benincasa@gsk.com (M.B.); miren.x.iturriza@gsk.com (M.I.); martina.x.carducci@gsk.com (M.C.); francesca.x.mancini@gsk.com (F.M.); rocio.x.canalsalvarez@gsk.com (R.C.); simona.x.rondini@gsk.com (S.R.)

**Keywords:** GMMA, serum bactericidal assay (SBA), nontyphoidal *Salmonella*, enteric fever, functional assay, antibodies, vaccine, iNTS disease

## Abstract

Salmonellosis remains a major cause of morbidity and mortality in low- and middle-income countries, despite the availability of effective vaccines against *Salmonella enterica* serovar Typhi (*S*. Typhi). In response, substantial efforts have been underway to develop vaccines against the key serovars responsible for invasive non-typhoidal *Salmonella* (iNTS) disease, such as *S*. Typhimurium and *S*. Enteritidis, as well as against *S*. Paratyphi A, which, together with *S*. Typhi, is responsible for enteric fever. The O-antigens (OAg) are considered potential protective antigens; therefore, the most advanced vaccine candidates focus on these moieties. However, no correlate of protection has been identified for either iNTS or paratyphoid fever, highlighting the importance of developing robust functional assays to assess vaccine-induced immunogenicity. In this study, we present the characterization of a high-throughput luminescence-based serum bactericidal assay (L-SBA) against multiple *S. enterica* serovars, using human sera. The assay was evaluated for repeatability, intermediate precision, linearity, and specificity against a panel of *Salmonella* strains belonging to serogroups O:4, O:9, and O:2, which were selected for their epidemiological relevance and diversity in OAg expression, quantity, and glucosylation/acetylation patterns. This assay will enable testing of clinical sera from vaccine trials to evaluate the breadth of the functional activity stimulated by current *Salmonella* vaccine candidates. L-SBA demonstrated an acceptable performance with all the tested strains, resulting in being linear, specific, and precise. This study also provided preliminary evidence that human sera containing antibodies against serogroup-specific OAg can efficiently kill *Salmonella* strains expressing OAg of the matched serovar, even in the presence of variation in OAg molecular weight, glucosylation, and acetylation. The L-SBA will enable testing of clinical sera from vaccine trials to evaluate the breadth of the functional activity stimulated by current *Salmonella* vaccine candidates.

## 1. Introduction

*Salmonella enterica* is a Gram-negative bacterium and a significant cause of foodborne illness worldwide, which is primarily transmitted through contaminated food and water. The burden of the disease is particularly relevant in low- and middle-income countries (LMICs), making it a global health concern [1,2,3,4]. *S. enterica* comprises six subspecies, each containing numerous serovars distinguished by their antigenic profiles. Among these, *S. enterica* subsp. *enterica* is the most medically relevant, encompassing serovars such as Typhi and Paratyphi A, which cause enteric fever—the most common bacterial bloodstream infection in South Asia [5,6,7,8]. It also includes serovars Typhimurium and Enteritidis, the major causes of bloodstream infections in sub-Saharan Africa, which are collectively referred to as invasive non-typhoidal *Salmonella* (iNTS) disease [9,10].

Despite the successful development of vaccines against *S*. Typhi, based on the Vi capsular antigen [11,12], no vaccine exists against *S*. Paratyphi A, Typhimurium, and Enteritidis.

Current efforts on developing vaccines against *S. enterica* serovars Typhimurium, Enteritidis, and Paratyphi A are focused on targeting the O-antigen (OAg) portion of the lipopolysaccharide. OAg consists of repeated sugar units with a backbone shared by many *Salmonella* serovars and specific sugars that define more restricted groups (abequose, tyvelose, and parathose for O:4, O:9, and O:2, respectively) and that can be further glucosylated and O-acetylated to create immunodominant, serovar-specific determinants. OAg has been found to mediate protective immunity in animal models [13,14,15,16], with anti-OAg antibodies able to mediate pathogen killing [13,14,17,18,19]. Multiple delivery platforms are being considered, such as glycoconjugates, GMMA (Generalized Module for Membrane Antigen), and MAPS (Multiple Antigen-Presenting System) [13,20,21,22,23,24,25,26,27], or other approaches such as live-attenuated bacteria [28,29].

In addition, several vaccine combinations against multiple serovars are currently being evaluated in Phase I and Phase II clinical trials. These include bivalent glycoconjugates, targeting *S*. Paratyphi A OAg and *S*. Typhi Vi capsular polysaccharides [30]; bivalent GMMA vaccines, targeting *S*. Typhimurium and *S.* Enteritidis OAg (ISRCTN51750695, PACTR202310834458532, and NCT06213506 [31]); and trivalent iNTS-TCVs. The latter either combines NTS GMMA with the licensed Typhoid Conjugate Vaccine (TCV) Vi-CRM197 (Typhibev manufactured by Biological E; NCT05480800), or combines two glycoconjugates, composed of *S*. Typhimurium and *S*. Enteritidis OAg, with another licensed TCV Vi-TT (Typbar manufactured by Bharat Biotech; NCT03981952, NCT05525546, and NCT05784701 [32]). A four-valent pan-*Salmonella* vaccine against *S*. Typhi, Paratyphi A, Typhimurium, and Enteritidis (preclinical phase [33]) is also being developed. All of these strategies rely on the principle that inducing sustained levels of anti-O:2, O:4, and O:9 antibodies, together with anti-Vi antibodies, can provide broader protection against *Salmonella* by targeting multiple serovars. The presence of antibodies that bind to the bacterial surface, along with evidence of their functional activity, represents a valuable indicator of potential vaccine-mediated protection [34,35,36]. To achieve this, a qualified high-throughput luminescence-based serum bactericidal assay (L-SBA) [37,38] is employed to analyze sera from vaccinated individuals. This assay has already proven to be valuable in analyzing preclinical sera and has provided relevant information on the immunogenicity of different vaccine platforms (GMMA vs. glycoconjugates) [27] and on the role of OAg fine specificities [39,40].

L-SBA was initially qualified to assess the functionality of antibodies against selected *S*. Typhimurium, *S*. Enteritidis [37], and *S*. Paratyphi A strains [38].

An in-depth phenotypic characterization of a large panel of *Salmonella* clinical isolates was performed [40], and a certain level of heterogeneity in terms of OAg expression level, molecular weight (MW) distribution, and acetylation and glucosylation levels was observed. It has been shown that *S*. Typhimurium and *S*. Enteritidis isolates expressed, under the same in vitro conditions, comparable OAg levels at the same optical density, while *S*. Derby expressed a lower amount. Most strains had a single OAg population with a medium MW ~20 kDa, and some strains presented a high-MW population at ~90 kDa. *S*. Typhimurium strains showed >80% O-acetylation, whereas *S*. Enteritidis strains and *S*. Derby had lower levels (8–28%), and *S*. Dublin showed ~60% O-acetylation. The majority of the strains of O:4 and O:9 serogroups had 13–23% glucosylation, but some *S*. Typhimurium strains reached 62% [40]. *S*. Paratyphi A strains presented a large variety of OAg expression levels among isolates, with most isolates displaying two main OAg populations, a medium-MW population around ~10 kDa and a high-MW population around ~90 kDa. O-acetylation ranged from 100% to as low as 17%. Glucosylation ranged from 49% to 95% [39]. Despite these differences in OAg characteristics, preclinical sera were able to induce sustained bactericidal activity against both homologous and heterologous strains belonging to the same serovar [39,40].

In preparation to analyze human samples from ongoing *Salmonella* vaccine trials, the SBA qualification was extended to include isolates representing epidemiologically relevant diversity [41,42,43,44,45,46] and covering a broad range of OAg characteristics. In detail, four isolates were selected for each one of the serogroups O:2, O:4 and O:9. The assay’s performance was evaluated using human standard reference sera with known reactivity against each OAg by providing evidence that OAg-induced antibodies, generated against a specific *Salmonella* strain, are capable of killing other clinical isolates of the same serovar, even with different OAg characteristics, at a level comparable to that obtained against the vaccine-homologous strains.

## 2. Materials and Methods

### 2.1. Bacterial Strains, Reagents, and Sera

All bacterial strains used in this study were selected for their epidemiological relevance and diversity in OAg glucosylation/acetylation patterns (as described in Table 1). Strains were stored frozen at −80 °C in 20% glycerol stocks and were grown at 37 °C in a Luria–Bertani (LB) medium, with stirring at 180 rpm. The overnight bacterial suspensions were then diluted in fresh LB to an optical density at 600 nm (OD600) of 0.05 and incubated at 37 °C at 180 rpm agitation in an orbital shaker until they reached the log phase (0.19–0.25 OD600) and then diluted to approximately 1 × 10^6^ Colony-Forming Units (CFUs)/mL in phosphate-buffered saline (PBS) [47]. Baby (3- to 4-week-old) rabbit complement (BRC), purchased from Cederlane, was stored in frozen aliquots and thawed immediately prior to use.

Two human reference standard sera were generated from subjects enrolled in two Phase I studies (NCT06213506 [31] and NCT05613205). Sera obtained 28 days after receiving two administrations of either *S*. Typhi-*S*. Paratyphi A or iNTS-TCV candidates or controls were screened by anti-OAg ELISA for anti-OAg IgG response. An equal volume of sera was pooled from subjects enrolled in NCT06213506 with anti-OAg IgG > 600 ELISA units (EUs)/mL against *S*. Typhimurium and *S*. Enteritidis (Std1) or ≥1000 EUs/mL against *S*. Paratyphi A from the NCT05613205 clinical study (Std2), respectively, to generate the two standards. Std1 was probed against all *S*. Typhimurium, *S*. Enteritidis, *S*. Derby, and *S*. Dublin strains, whereas Std2 was probed against *S*. Paratyphi A strains.

Working aliquots of the human standard sera were stored at −80 °C until use. Aliquots were heat-inactivated (HI) at 56 °C for 30 min to remove endogenous complement activity before using them in SBA.

Repeatability and intermediate precision: Twelve identical standard sera (independent dilutions) were assayed by the same operator for four different days, independently (forty-eight samples in total). Statistical analysis was performed by Minitab v22 (Minitab Inc., Chicago, IL, USA). A Mixed-Effects Model was used, considering the day as random and no fixed factor, reporting total variability of the method, the intermediate precision, and the repeatability on LogIC50-transformed values to ensure normality of the data distribution. All data were used except one outlier in the case of *S*. Enteritidis A1636 and three others in the case of *S*. Dublin ATCC39184, as determined by outlier analysis. Outlier analysis was performed using the ROUT method by GraphPad Prism 7 software.

Linearity: Standard sera were assayed neat or diluted in PBS in 2-fold steps at 1:2, 1:4, 1:8, 1:16, and 1:32. Correlation was calculated between observed Log_10_-transformed IC50 and the Log_10_-nominal IC50 divided by the dilution factor, and R^2^ was obtained together with confidence intervals (CIs).

Limit of detection and limit of quantification: Standard sera were diluted in PBS to generate a sample with a low, but detectable, SBA titer (expected IC50 around 100). Twelve identical samples were assayed on the same day by the same operator.

Specificity: Standard sera were diluted 1:1 (*v:v*) in PBS, supplemented with different quantities of homologous or heterologous antigens at different final concentrations (100, 50, 25, 10, and 1 µg/mL antigen) and compared with a sample spiked 1:1 (*v:v*) with PBS alone. All samples were incubated overnight (16–18 h) at 4 °C prior to being tested. The lowest concentration of a homologous competitor able to inhibit ≥70% the IC50 of the PBS-spiked samples for each strain was subsequently used in a second experiment to determine the heterologous specificity. For the heterologous specificity assessment, human standard sera diluted 1:1 (*v:v*) in PBS supplemented with GMMA carrying *Salmonella* O:4, O:9, and O:2, *Shigella flexneri* 1b OAg, or Vi antigen at the specific concentration determined in the initial assessment for each strain were assayed and compared with samples preincubated overnight with an equal volume of PBS alone (undepleted). The percentage of inhibition was determined by calculating the decrease in the observed SBA titers between samples pre-treated with a competitor and the undepleted control.

### 2.2. Luminescent SBA (L-SBA)

**Figure 1:**
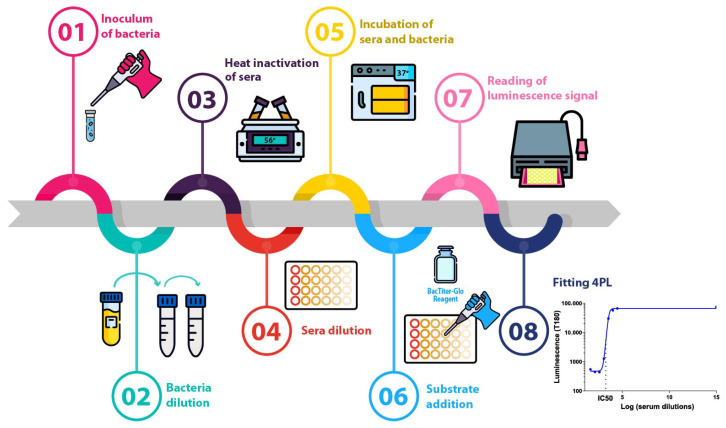
L-SBA flowchart procedure. Main steps to measure the bactericidal activity of antibodies elicited by *S*. Typhi-*S.* Paratyphi A and iNTS-TCV candidates.

SBA based on luminescent readout (L-SBA) was performed, adapting the same protocol used to determine functionality against vaccine-homologous strains [37,38], as reported in Figure 1. Briefly, a 3-fold dilution step up to 7 dilution points of HI test sera and 1 control well with no sera were incubated in the presence of exogenous complement (BRC, 50% for all *S*. Typhimurium and Enteritidis strains, 5% for *S*. Derby, 15% for *S*. Dublin, and 20% for all *S*. Paratyphi A strains) and bacteria (approximately 2 × 10^4^ CFUs/mL from log-phase cultures stopped at OD600 = 0.22 ± 0.03 [51]) in a final reaction volume of 100 µL/well. The starting dilution of each serum in the assay was adapted on the basis of parameters assayed: 1:20 for all *S*. Paratyphi A strains, 1:100 for *S*. Derby and for all *S*. Typhimurium strains tested, 1:500 for *S*. Dublin, 1:1000 for *S*. Enteritidis A1636 and D7795, 1:2000 for *S*. Enteritidis CMCC4314 in case of repeatability and intermediate precision, linearity, and homologous and heterologous precision assessment, and 1:4 for all strains in case of limit of detection and limit of quantification assessment.

The final reaction mixture was mixed and incubated for 3 h at 37 °C (T180). At the end of the incubation, the SBA plate was centrifuged at room temperature for 10 min at 4000× *g*. The supernatant was discarded, and the bacterial pellets were resuspended in 100 µL of PBS, transferred to a white round-bottom 96-well plate (Greiner), and mixed in a 1:1 (*v:v*) ratio with BacTiter-Glo Reagent (Promega, Southampton, UK). The reaction was incubated for 5 min at room temperature on an orbital shaker, and the luminescence signal was measured by a luminometer (Synergy HT, Biotek, Swindon, UK).

A 4-parameter non-linear regression was applied to the raw luminescence (no normalization of the data was applied) obtained for all the serum dilutions tested for each serum; an arbitrary serum dilution of 10^15^ was assigned to the well containing no serum. Fitting was performed, constraining the curves to have a bottom between 0 and below a value equal to the average value of the luminescence detected at T180 for standard sera in wells in which bacteria are killed [52]. The constraining value was calculated for all the repeats of sera tested for repeatability and intermediate precision evaluation. The results of the assay are expressed as IC50, represented by the reciprocal serum dilution that results in a 50% reduction in luminescence. GraphPad Prism 7 software (GraphPad Software, La Jolla, CA, USA) was used for fitting and IC50 determination.

## 3. Results

In preparation to assess the bactericidal activity of vaccine-induced antibodies against *Salmonella*, a panel of epidemiologically relevant clinical isolates with different OAg acetylation/glucosylation profiles belonging to serogroups O:2, O:4, and O:9 [41,42,43,44,45,46] was selected, and the performance of the L-SBA was evaluated. In particular, for serogroup O:2, four *S*. Paratyphi A isolates with acetylation ranging from 17 to 100% and glucosylation from 51 to 20% were selected; for serogroup O:9, three *S*. Enteritidis isolates and one *S*. Dublin, with O-acetylation ranging from 8 to 60% and glucosylation from 13 to 21%; for serogroup O:4, three *S*. Typhimurium isolates with O-acetylation around 120% and glucosylation ranging from 18 to 62% and *S*. Derby, with O-acetylation <28% and a glucosylation of 18% (Table 1).

### 3.1. Precision, Limit of Detection, and Limit of Quantification

**Figure 2:**
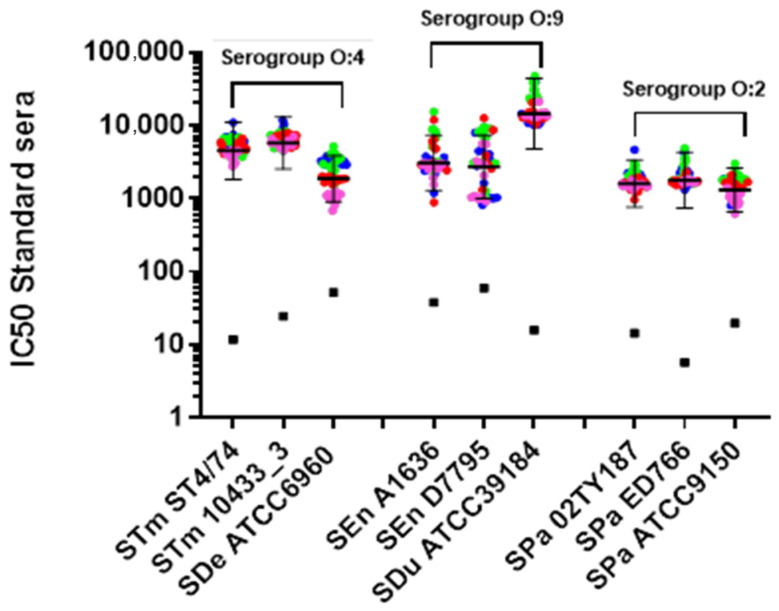
Assay precision for strains belonging to O:4, O:9, and O:2 serogroups. A total of 48 repeated measurements of IC50 for each strain from single independently handled samples, by one operator on four different days, is shown. Repeats of each operator are represented by circle symbols (repeats on different days are shown in blue for day 1, green for day 2, red for day 3, and pink for day 4). Geometric means and 95% confidence intervals from 48 repeats are represented by the black lines and error bars for each of the tested samples. Black square symbols indicate the limit of quantification.

The precision of the assay was determined by testing the two human standards multiple times across multiple days against each *S. enterica* isolate (Figure 2). Precision against vaccine-homologous strains was previously assessed and therefore not repeated.

Repeatability was <4% coefficient of variation (CV) on Log_10_-transformed data for most of the strains. *S*. Enteritidis showed the highest variability, with repeatability values up to 9.5% CV. Intermediate precision was <10.4% CV across all strains (Table 2). No significant day-to-day effect on assay variability was observed for any strain (*p*-value > 0.05).

The limit of detection and the limit of quantification of the assay were ≤10 and <60 IC50, respectively (Table 3), confirming that the assay can accurately quantify serum functional activity even at low reactivity levels.

### 3.2. Linearity

Linearity of all assays was confirmed, as the 95% CI of the intercepts of IC50 values, obtained for each sample as a function of the initial dilution, did not differ significantly from 0, and slopes did not differ significantly from 1 for all samples (Table 4). Linearity of assays against vaccine-homologous strains was previously assessed and therefore not repeated.

### 3.3. Specificity

The specificity of the assay was determined by inhibition experiments performed by depleting human standard sera with varying amounts of homologous or heterologous OAg prior to testing bactericidal activity in SBA against each strain, and this was compared to the killing induced by undepleted sera. The capsular Vi polysaccharide was also included as a competitor, as this antigen is present in most *Salmonella* vaccine formulations that are currently in clinical testing. For each assay, the concentration of competitors was set at the lowest serogroup-matched OAg concentration capable of inhibiting ≥70% of the bactericidal activity. This was found to be 10 µg/mL for all *S*. Typhimurium strains, *S.* Enteritidis strains A1636 and D7795, and *S*. Dublin ATCC39184; 1000 µg/mL for *S*. Derby ATCC6960; 100 µg/mL for *S*. Enteritidis CMCC4314; and 50 µg/mL for *S*. Paratyphi A strains.

**Figure 3:**
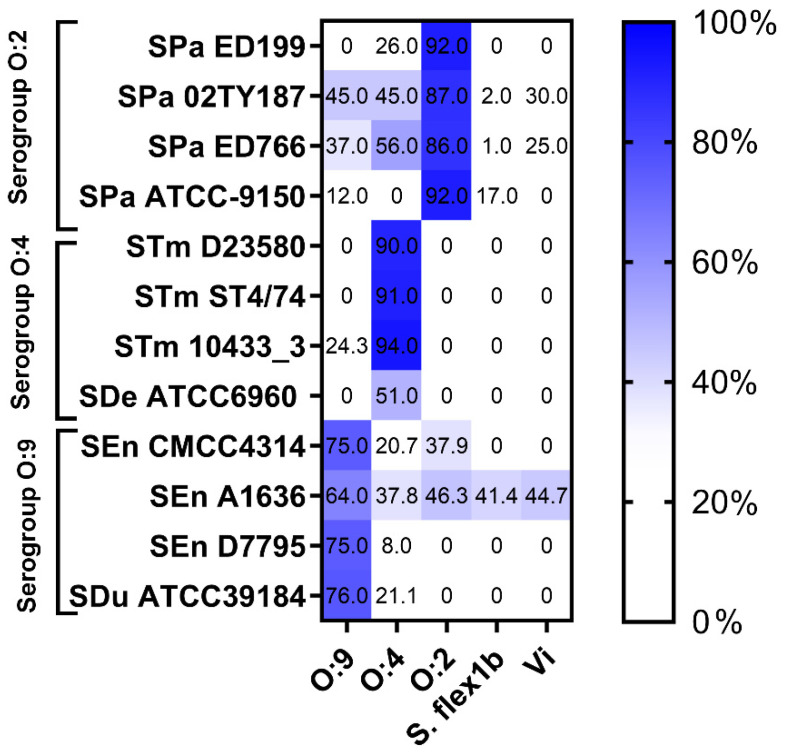
Heatmap of IC50 inhibition (%) with purified polysaccharides for all strains tested: the percentage of IC50 inhibition observed after depletion with a specific polysaccharide was calculated compared to the control spiked with an equal amount of PBS.

Depletion with serogroup-matched OAg resulted in ≥70% inhibition of IC50 in 10 out of 12 cases, confirming that the assay can effectively determine the functionality of antibodies directed against serogroup-matched OAg. Only in the cases of *S*. Derby ATCC6960 and *S*. Enteritidis A1636 was the percentage of inhibition with matched OAg below 70% (Figure 3).

Depletion with heterologous OAg, either from *Shigella flexneri* 1b or from a different *Salmonella* serogroup, resulted in <30% inhibition of IC50 for most of the strains tested.

SBA against *S*. Enteritidis (strains CMCC4314 and A1636) and *S*. Paratyphi A (strains 02TY187 and ED766) also showed some partial inhibition when sera were spiked with heterologous polysaccharides, possibly indicating some OAg cross-reactivity.

Overall, these data indicate good specificity for the SBAs and suggest that antibodies targeting serogroup-matched OAg are primarily responsible for the bactericidal activity observed.

## 4. Discussion and Conclusions

Enteric fever and iNTS disease caused by *S. enterica* serovars Typhi, Paratyphi A, Typhimurium, and Enteritidis represent significant public health concerns, especially in LMICs. Despite the successful introduction of vaccines against *Salmonella* Typhi, there is still no vaccine against these other *Salmonella* serovars, and this has become a WHO priority [53]. Consequently, considerable efforts are underway to develop vaccines against these pathogens. Combination vaccines are considered an ideal solution to target multiple *Salmonella* serovars, and different candidates are currently under clinical evaluation.

No correlate of protection exists for these pathogens; therefore, functional assays, together with traditional binding assays, are considered a useful tool to better characterize the immune response elicited by *Salmonella* vaccines under development. In addition, by testing epidemiologically relevant clinical isolates, some indications can be obtained on the ability of a specific vaccine to protect across multiple strains. Among functional assays, the relevance of SBA as a key readout has been increasingly recognized. This is supported by evidence showing that age-related acquisition of O-antigen-specific SBA parallels the declining incidence of iNTS [54,55], which is consistent with a role for naturally acquired protective immunity.

Furthermore, maternal anti-LPS antibodies that confer protection to infants are associated with delayed NTS seroconversion, further indicating that antibody-mediated bactericidal activity contributes to early-life protection and shapes subsequent age-dependent susceptibility [56].

A high-throughput L-SBA method to determine the bactericidal activity in both preclinical [51] and clinical sera [52] was developed, and recent work was performed with sera from immunized animals, showing that multiple strains, belonging to the same serovar, can be killed, despite differences in their OAg-specific characteristics [39,40]. Demonstrating that a vaccine could theoretically protect against multiple clinical isolates, regardless of their OAg features, is important to support further clinical development, and, to our knowledge, similar information is not yet broadly available for any of the vaccines in clinical development.

Therefore, with the aim of confirming these preclinical results using clinical sera from ongoing vaccine trials, the qualification of this L-SBA [37,38] was extended. The assay, originally qualified with one strain per serovar, was expanded to include additional *Salmonella* strains exhibiting differences in OAg characteristics.

L-SBA demonstrated an acceptable performance with all the tested strains, similar to what was previously reported [37,38], and therefore, it was suitable to test clinical samples. The variability observed was highest for *S*. Enteritidis and lowest for *S.* Paratyphi A strains, for which intermediate precision and repeatability were consistently ≤5%. The assay was linear and specific to assess the activity of antibodies targeting serogroup-matched OAg, and day-to-day variation did not significantly impact its overall variability.

Depletion experiments using different polysaccharides demonstrated that the bactericidal activity was predominantly driven by serogroup-specific anti-OAg antibodies, with minimal killing attributed to unrelated OAg—such as that observed after depletion with *S. flexneri* 1b OAg—or the Vi capsular polysaccharide.

Interestingly, the specificity analysis using serogroup-unmatched OAg provided additional insights into possible cross-reactivity, which seemed more pronounced for certain serovars. Such a pattern could not be readily explained by the specific OAg characteristics described here (molecular weight, O-acetylation, and glucosylation levels), nor can it be simply assigned to the presence of the common O:12 backbone, which is shared across all OAg variants. Another factor that may contribute to cross-reactivity in these depletion assays is the functional activity of antibodies directed against *Salmonella* proteins [49,57], some of which are conserved across strains and could therefore mediate bactericidal activity, independently of OAg specificity. It should be noted that this analysis was limited by the use of polyclonal sera and by depletion experiments performed only with antigens containing the vaccine-homologous polysaccharide. A more detailed investigation could use monoclonal antibodies targeting defined OAg features, combined with depletion using OAg variants that differ in specific attributes. Such experiments were beyond the scope of the present study but could be addressed in future work.

Assay standardization is critical, as multiple vaccine candidates, using different OAg delivery platforms, are in clinical development. For example, in a preclinical study [27], GMMA-induced antibodies demonstrated higher functional activity than traditional conjugates, despite eliciting similar total antibody levels. This result may reflect variations in antibody quality rather than quantity. To date, a clinical head-to-head comparison between conjugate and GMMA platforms has not been attempted; however, as additional clinical programs progress into clinical phases, indirect comparison of functional responses across platforms will become increasingly feasible.

Meaningful cross-platform comparisons will require standardized assay reagents and conditions and harmonized functional assay protocols. Parameters such as incubation time, the percentage of exogenous complement used, and the inoculum growth phase can all influence the measured bactericidal activity; therefore, they must be harmonized to ensure interstudy comparability.

The standardized assay described here will facilitate the development of multicenter proficiency panels for *Salmonella* SBAs performed under uniform conditions, enabling more reliable cross-study comparisons.

In addition, this standardized method can support the identification of correlates of protection and the definition of protective thresholds. Studies have already been planned to test sera collected from human challenge studies and vaccine trials and to compare their reactivity profiles with those from sero-epidemiological studies.

In conclusion, this work demonstrates that human sera containing anti-OAg antibodies can efficiently kill *Salmonella* strains expressing OAg of the matched serovar, even in the presence of variation in OAg glucosylation and acetylation or other structural modifications. These findings increase confidence that candidate vaccines currently in development may induce broad cross-protection against circulating *Salmonella* strains.

## Figures and Tables

**Table 1:** Bacterial strains used in this study.

Serogroup	Strain/Isolate ID	Strain/Isolate ID (Abbreviated)	OAg Features (* Oac %) [Ref]	OAg Features (* Glc %) [Ref]
O:2	*S*. Paratyphi A ED199	SPa ED199	61 [39]	78 [39]
O:2	*S*. Paratyphi A 02TY187	SPa 02TY187	54 [39]	51 [39]
O:2	*S*. Paratyphi A ED766	SPa ED766	100 [39]	79 [39]
O:2	*S*. Paratyphi A ATCC9150	SPa ATCC9150	17 [39]	57 [39]
O:4	*S*. Typhimurium D23580	STm D23580	129 [40]	18 [40]
O:4	*S*. Typhimurium ST4/74	STm ST4/74	118 [40]	62 [40]
O:4	*S*. Typhimurium 10433_3	STm 10433_3	120 [40]	39 [40]
O:9	*S*. Enteritidis CMCC4314	SEn CMCC4314	<8 [40]	16 [40]
O:9	*S*. Enteritidis A1636	SEn A1636	28 [40]	13 [40]
O:9	*S*. Enteritidis D7795	SEn D7795	23 [40]	21 [40]
O:4	*S*. Derby ATCC6960	SDe ATCC6960	<28 [40]	18 [40]
O:9	*S*. Dublin ATCC39184	SDu ATCC39184	60 [40]	18 [40]

* Oac %: OAg acetylation percentage; Glc %: OAg glucosylation percentage. GMMA containing penta-acylated lipid A was purified from *S*. Typhimurium, *S*. Enteritidis, and *S*. Paratyphi A mutants [47,48,49]. The OAg was extracted from *S*. Typhimurium, *S*. Enteritidis, and *S*. Paratyphi A GMMA by direct acid hydrolysis. The OAg was fully characterized in terms of sugar content, protein, and nucleic acid impurities, as previously reported [50].

**Table 2:** Repeatability and intermediate precision (CV% on Log10-transformed data).

	Serogroup O:4			Serogroup O:9			Serogroup O:2		
	STm ST4/74	STm 10433_3	SDe ATCC6960	SEn A1636	SEn D7795	SDu ATCC39184	SPa 02TY187	SPa ED766	SPa ATCC9150
Repeatability (CV% on Log10-transformed data)	2.20	2.22	3.35	6.06	9.46	1.86	2.73	3.12	3.03
Intermediate Precision (CV% on Log10-transformed data)	3.32	2.44	7.43	7.14	10.37	4.63	3.90	3.84	4.84

**Table 3:** Limit of detection (LoD) and limit of quantification (LoQ).

Serogroup O:4				
Strain	STm D23580	STm ST4/74	STm 10433_3	SDe ATCC6960
LoD (IC50)	4.7	5.7	7.3	9.3
LoQ (IC50)	6.4	11.7	24.2	51.7
Serogroup O:9				
Strain	SEn CMCC4314	SEn A1636	SEn D7795	SDu ATCC39184
LoD (IC50)	10.0	8.4	9.8	6.3
LoQ (IC50)	63.5	37.7	59.5	15.7
Serogroup O:2				
Strain	SPa ED199	SPa 02TY187	SPa ED766	SPa ATCC9150
LoD (IC50)	5.4	6.1	4.5	6.8
LoQ (IC50)	10.1	14.3	5.7	19.7

**Table 4:** Coefficients of regression analysis for each of the tested *Salmonella* strains.

		Slope (CI 95% Limits)	Intercept (CI 95% Limits)
Serogroup O:4	STm ST4/74	1.08	−0.04
		(0.91; 1.25)	(−0.91; 0.15)
	STm 10433_3	1.07	−0.02
		(0.99; 1.14)	(−0.42; 0.01)
	SDe ATCC6960	1.03	−0.20
		(0.86; 1.19)	(−0.75; 0.35)
Serogroup O:9	SEn A1636	0.94	−0.02
		(0.47; 1.41)	(−1.32; 1.3)
	SEn D7795	0.71	0.76
		(−0.02; 1.4)	(−1.39; 2.89)
	SDu ATCC39184	1.01	−0.06
		(0.74; 1.28)	(−0.97; 0.84)
Serogroup O:2	SPa 02TY187	0.95	0.08
		(0.78; 1.12)	(−0.31; 0.47)
	SPa ED766	0.94	0.18
		(0.84; 1.05)	(−0.08; 0.45)
	SPa ATCC9150	1.08	−0.23
		(0.97; 1.19)	(−0.51; 0.04)

## Data Availability

The original contributions presented in this study are included in the article. Further inquiries can be directed to the corresponding author.
